# Comprehensive Evaluation and Application of a Novel Method to Isolate Cell-Free DNA Derived From Bile of Biliary Tract Cancer Patients

**DOI:** 10.3389/fonc.2022.891917

**Published:** 2022-05-04

**Authors:** Ningjia Shen, Bin Zhu, Wei Zhang, Baoning Nian, Xiaoya Xu, Lianghe Yu, Xiang Ruan, Sheng Chen, Yang Liu, Xinkai Cao, Xintong Shi, Zhikuan Li, Xingfeng Huang, Xiang Wang, Caifu Chen, Lei Xiong, Dadong Zhang, Xiaohui Fu, Yongjie Zhang

**Affiliations:** ^1^ Department of Biliary Surgery, Shanghai Eastern Hepatobiliary Surgery Hospital, Second Military Medical University/Navy Medical University, Shanghai, China; ^2^ 3D Medicines Inc., Shanghai, China

**Keywords:** bile, cell-free DNA, extraction, yield, liquid biopsy

## Abstract

Cell-free DNA (cfDNA) exists in various types of bodily fluids, including plasma, urine, bile, and others. Bile cfDNA could serve as a promising liquid biopsy for biliary tract cancer (BTC) patients, as bile directly contacts tumors in the biliary tract system. However, there is no commercial kit or widely acknowledged method for bile cfDNA extraction. In this study, we established a silica-membrane-based method, namely 3D-BCF, for bile cfDNA isolation, exhibiting effective recovery of DNA fragments in the spike-in assay. We then compared the 3D-BCF method with four other commercial kits: the BIOG cfDNA Easy Kit (BIOG), QIAamp DNA Mini Kit (Qiagen), MagMAX^TM^ Cell-Free DNA Isolation Kit (Thermo Fisher), and NORGEN Urine Cell-Free Circulating DNA Purification Mini Kit (Norgen Biotek). The proposed 3D-BCF method exhibited the highest cfDNA isolation efficiency (p < 0.0001) from patient bile samples, and bile cfDNA of short, medium or long fragments could all be extracted effectively. To test whether the extracted bile cfDNA from patients carries tumor-related genomic information, we performed next-generation sequencing on the cfDNA and verified the gene-mutation results by polymerase chain reaction (PCR)-Sanger chromatograms and copy-number-variation (CNV) detection by fluorescence *in situ* hybridization (FISH) of tumor tissues. The 3D-BCF method could efficiently extract cfDNA from bile samples, providing technical support for bile cfDNA as a promising liquid biopsy for BTC patient diagnosis and prognosis.

## Introduction

Liquid biopsy has attracted increasing attention owing to its broad perspectives in cancer diagnosis and monitoring prognosis (1). The majority of liquid biopsy techniques have focused on cell-free DNA (cfDNA), which is released from cells and could be detected in various types of bodily fluids (2). cfDNA originates from cell autonomous release, apoptosis or necrosis, and circulating tumor cells (CTCs) dissociation (3). Compared to traditional tissue biopsy, cfDNA is more accessible and able to avoid tumor heterogeneity, thus providing more accurate information of cancer genomes with minimal invasiveness and much greater convenience (4).

While plasma cfDNA is the most widely investigated, research on urine, cerebrospinal fluid, effusion, and other bodily fluids has also been reported (2, 5, 6). However, blood flows through the entire body, causing tumor-originated cfDNA to attenuate in the circulatory system, which may result in more difficulty in detection. Other bodily fluids also contain cfDNA and may even outperform plasma cfDNA in the detection of some specific malignancies, such as urine cfDNA in bladder cancer and cerebrospinal fluid cfDNA in glioma (7–9). Bile, secreted mostly by hepatocellular cells, flows within the biliary tract and is stored in the gallbladder, and maintains direct contact with biliary tract system. Thus, it may be a better source of tumor markers for detecting biliary tract and other gastrointestinal cancers (10). In patients with biliary tract cancers (BTCs), bile cfDNA exhibited better sensitivity and specificity in the detection of gene mutations (94.7% and 99.9%, respectively) than plasma cfDNA, indicating its potential advantage in BTC diagnosis (11–13). Furthermore, bile cfDNA fragments are much longer than the highly fragmented plasma cfDNA, which may be due to the extreme physiochemical condition of bile, indicating that bile cfDNA may carry more information about genome aberration (12). For patients with hepatobiliary lesions who are prone to cholestasis, bile must be drained to avoid jaundice; thus, it is worth investigating whether bile could be applied to early diagnosis of hepatobiliary malignancies. However, there is no commercial cfDNA extraction kit available for bile, so it is urgent to establish a method for efficiently extracting bile cfDNA of high quality.

The characteristics of cfDNA fragments are one major consideration for cfDNA extraction. For example, the main peak of cfDNA from plasma or serum is 167-168 bp (14). Referring to this segment feature of cfDNA from plasma or serum, the commercial kits, including the QIAamp Circulating Nucleic Acid Kit (Qiagen) among others were originally designed for the purification of highly fragmented cfDNA from plasma or serum, and capturing DNA fragments longer than 75 bps according to the manufacturer (15). QIAamp Circulating Nucleic Acid Kit (Qiagen) is widely used to isolate bile cfDNA (11–13, 16), however, it was found in our previous study and that of Arechederra M et al. that long fragments of cfDNA (> 6,000 bp) were prevalent in bile (12, 17), suggesting it was required to establish the extraction method of bile cfDNA considering its fragmentation feature.

In this study, we constructed a method for extracting bile cfDNA with a silica membrane that is capable of capturing DNA fragments as short as 40 bps and as long as 10 kb, namely, 3D-BCF. To verify its ability to extract DNA from bile, we performed a spike-in assay by mixing artificial DNA fragments of different concentrations and lengths into the bile, and then calculated the recovery rate of the DNA fragments of each length. We further assessed the ability of extracting cfDNA from patient bile samples and the quality of extracted bile cfDNA by comparing our extraction method (3D-BCF) to four other commercial kits, namely the BIOG cfDNA Easy Kit (BIOG), QIAamp DNA Mini Kit (Qiagen), MagMAX^TM^ Cell-Free DNA Isolation Kit (Thermo Fisher), and NORGEN Urine Cell-Free Circulating DNA Purification Mini Kit (Norgen Biotek). Finally, we performed next-generation sequencing on the extracted bile cfDNA and verified mutations by Sanger chromatograms and CNVs by FISH. In general, we established a method suitable for bile cfDNA extraction and a quality-control system of bile cfDNA, providing technical support for bile cfDNA as a new way of conducting tumor liquid biopsies.

## Materials and Methods

### Clinical Samples and Pretreatment

Bile samples from four patients with malignant biliary tumors were collected from August 2017 to March 2018; all bile samples were collected prior to surgery. The bile samples were collected in BEAVER cell free DNA tubes (BEAVER, China) and centrifuged at 1,600 g at 4°C for 10 min followed by another 16,000 g at 4°C for 15 min to remove cell debris. Subsequently, the supernatant was removed and stored in a refrigerator at −80°C for nucleic acid extraction. A formalin-fixed paraffin-embedded (FFPE) tissue sample of one of the BTC patients was obtained from the clinical sample bank at the Department of Biliary II, Shanghai Eastern Hepatobiliary Surgery Hospital, Navy Military Medical University. To ensure that the content of cancer cells in FFPE specimens was ≥ 60%, the patients’ status was confirmed by surgical pathology and the section samples reviewed by a pathologist. This study was approved by the Ethics Committee of Shanghai Eastern Hepatobiliary Surgery Hospital of Naval Military Medical University (Second Military Medical University) (EHBHKY2018-K-003), and all patients signed a written informed consent form before patient samples and clinical information were used.

### Establishment of 3D-BCF Bile cfDNA Extraction Kit (3D-BCF)

**Figure 1:**
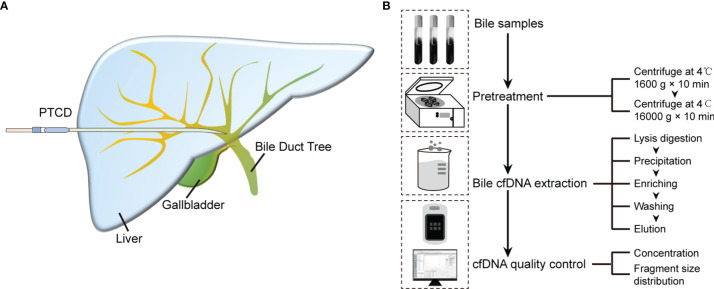
Bile cfDNA extraction procedure and cfDNA quality control of 3D-BCF method. **(A)** Schematic diagram of bile sample collection. **(B)** Flowchart of bile cfDNA extraction and quality control.

The process of the 3D-BCF method established in this study mainly includes lysis of bile samples, precipitation, enrichment, and washing and elution of bile cfDNA. A schematic of the experimental layout is provided in **Figure 1**. The detailed process is the following.

1) Lysis: 240 μL of buffer ACL (Cat. No. 939017, Qiagen) and 1.68 μL of carrier RNA (Cat. No. 1017647, Qiagen) were mixed, added to 300 μL of bile, and vortexed. A total of 30 μL of proteinase K (Cat. No. 19133, Qiagen) was added, and then the solution was shaken upside down and incubated at 60°C for 30 min. During incubation, it was mixed upside down approximately every 10 min.

2) Precipitation: When the incubation was completed, 540 μL of buffer ACB (Cat. No. 1069275, Qiagen) was added to the above compound, which was placed in a refrigerator at −20°C for 5 min after vortexing.

3) Enrichment: In total, 700 μL of the mixture was pipetted and added to the QIA-quick column (Cat. No. 28115, Qiagen). When loading it for the first time, the column was incubated at room temperature for 5 min. After incubation, the mixture was centrifuged at 6,000 rpm for 1 min, and then the remaining mixture was then passed through an adsorption column under the same centrifugation conditions.

4) Washing: The aforementioned column was filled with 500 μL of buffer PE (Cat. No. 19065, Qiagen) and centrifuged at 6,000 rpm for 1 min; this washing step was repeated once. The mixture was then centrifuged at 13,000 rpm for 1 min to remove any remaining liquid in the column.

5) Elution: In total, 30 μL of elution buffer (Cat. No. 19086, Qiagen) was pipetted into the column, and left at room temperature for 3 min; it was then centrifuged at 13,000 rpm for 1 min to elute and store cfDNA.

### Recovery Efficiency of 3D-BCF Method for Bile cfDNA

For the spike-in assay, the solvent was the bile sample collected from one BTC patient. Five DNA fragments of different sizes [100, 600, 1,000, 6,000 and 8,000 bps (Thermo Fisher)] were mixed at equal mass ratios, and the fragment mixture of 0, 300, 900, and 1,500 ng was added to 300 μL of patient bile samples respectively. There were four groups in total, with three repetitions in each group. The patient’s bile sample itself was verified by the 3D-BCF method to have very low cfDNA content. The 3D-BCF method was then used to extract spike-in cfDNA and detect the concentration and fragment-size distribution. Using 2100 Expert software to analyze and detect the recovery efficiency of each fragment size, we defined the area under the curve of fragment size ranges [95 bp, 105 bp], [580 bp, 640 bp], [922 bp, 1,157 bp], [4,545 bp, 6,162 bp], and [6,162 bp, 8,385 bp] as the content of fragment 100 bps, 600 bps, 1,000 bps, 6,000 bps and 8,000 bps according to the positive control (the peak shape displayed by the unextracted spiked-in DNA). The content was recorded as A, and the 0-ng group was used as a negative control. The recovery efficiency of a certain fragment is


$$\text{N(\%)}=\frac{\bar{A_{sample}}\;-\;\bar{A_{nagetive}}}{\bar{A_{positive}}}\times 100\%$$

where $\bar{A_{sample}}$ is the average value of a fragment size content of the sample, and $\bar{A_{positive}}$ and $\bar{A_{nagetive}}$ are the average content of the positive and negative control, respectively, corresponding to the size of the fragment.

### Bile cfDNA Extraction, Concentration Measurement, and Fragment-Size-Distribution Detection

Three bile samples were selected from patients with malignant biliary tumors in terms of total bile cfDNA, purity, and fragment-size distribution by comparing the extraction using the 3D-BCF method with that using four other nucleic acid extraction kits. Each patient’s bile was divided into 15 aliquots, and three aliquots were extracted by each method and recorded as three repetitions. Subsequently, five methods were used to isolate the bile cfDNA, among which the 3D-BCF method was as described above, and the four kits were implemented in accordance with the manufacturer’s instructions. The procedures, in brief, were as follows.

BIOG cfDNA Easy Kit (Cat. No. 51019, BIOG; abbreviated as BIOG): 6 μL of DNA carrier was added to 300 μL of bile sample and vortex, and then filled with 450 μL of lysis buffer and 30 μL of digestion solution. The mixture was then turned upside down and incubated at 56°C for 10 min. One milliliter of ethanol was added to the mixture, vortexed to transfer 760 μL to the column, and then centrifuged at 12,000 rpm at 4°C for 1 min. This centrifugation step was repeated until the remaining sample passed through the column. In total, 500 μL of washing solution A was added to the column and centrifuged at 12,000 rpm for 1 min at 4°C. The same procedure was performed using washing solution B, and then the column was centrifuged at 12,000 rpm for 1 min. Finally, 30 μL of elution buffer was transferred to the column, and the cfDNA stored after centrifugation at 12,000 rpm at 4°C for 2 min.

QIAamp DNA Mini Kit (Cat. No. 51304, Qiagen; abbreviated as QIA-mini): 180 μL of buffer ATL and 20 μL of PK was added to 300 μL of bile, and then mixed well by vortexing and incubated at 56°C for 1 h. Then, 200 μL of buffer AL was added, mixed by vortexing, and incubated at 70°C for 10 min. After incubation, 200 μL of ethanol was added and mixed well. The mixture was then added to the column and centrifuged at 6,000 g for 1 min; this centrifugation step was repeated until all remaining samples passed through the column. Subsequently, 500 μL of buffer AW1 was pipetted into the column and centrifuged at 6,000 g for 1 min. The filtrate was discarded and 500 μL of buffer AW2 added, centrifuged at 21,000 g for 3 min, and then centrifuged again at 21,000 g for 1 min. Finally, 30 μL of buffer AE was added and the mixture centrifuged at 6,000 g for 1 min to elute and store the cfDNA.

MagMAX™ Cell-Free DNA Isolation Kit (Cat. No. A29319, Thermo Fisher; abbreviated as MagMAX): 6 μL proteinase K was added to 300 μL of bile and mixed upside down; then, 15 μL 20% SDS was added and the mixture incubated at 60°C for 20 min. After incubation, the mixture was placed on ice for 5 min. After returning to room temperature, the solution and magnetic beads mixture, composed of 300 μL of MagMAX™ cell free DNA lysis/binding solution and 6 μL of MagMAX™ cell free DNA magnetic beads, was added. The mixture was then shaken at medium speed on a vortex mixer for 10 min, and placed in a magnetic stand for 5 min after instant separation until the solution was clear. The supernatant was removed, 500 μL of MagMAX™ cell free DNA wash solution was added, and the solution was mixed well. It was placed in the magnetic stand again until it was clear, and then the supernatant was discarded. After washing twice with 500 μL of 80% ethanol and drying on a magnetic stand at room temperature, 30 μL of elution buffer was finally pipetted into it and the resulting mixture placed on a magnetic stand for 2 min until the solution was clear. The supernatant was aspirated and the cfDNA was stored.

Urine Cell-Free Circulating DNA Purification Mini Kit (Cat. No. 56800, Norgen Biotek; abbreviated as NORGEN): 300 μL of bile sample was diluted to 2 mL with PBS, 20 μL of proteinase K was added, and the solution was mixed well by vortexing and incubation at 55°C. After incubation, 400 μL of binding solution K was added and the solution mixed thoroughly. A 800-μL sample was transferred to a Mini Spin column, centrifuged at 3,300 g for 2 min, and all remaining samples were dropped through the column under the same centrifugal conditions. Then, 600 μL of wash solution A was added and the mixture centrifuged at 3,300 g for 1 min. It was centrifuged again at 14,000 g for 2 min; 30 μL of elution buffer B was added to the column, the mixture was centrifuged at 200 g for 1 minute, and then at 5,200 g for 2 minutes to collect and store cfDNA.

After the bile cfDNA was extracted by the above methods, a NanoDrop 2000 (Thermo Fisher), Qubit 3.0 fluorometer (Thermo Fisher), and Agilent 2100 bioanalyzer (Agilent Technologies, Inc.) were used to detect the cfDNA purity, concentration, and fragment distribution of cfDNA, respectively.

### Next-Generation Sequencing to Detect Gene Mutation Signals of bile cfDNA

The bile cfDNA fragments are mainly long fragments, and the fragmentation must be performed with a Covaris S2 Sonolab (Covaris, Inc.) before constructing the sequencing library; with the fragment size of approximately 200–400 bps. The cfDNA targeted deep sequencing platform with the unique identification (UID) indexed capturing-based sequencing (UC-Seq) (doi: 10.1186/s12885-018-4199-7) was used for a 733 gene panel. Finally, the sequencing data were analyzed by bioinformatics and the cutoff value of mutant allele frequencies (MAFs) in bile cfDNA determined to be ≥ 0.01.

### Sanger Sequencing Detection of SNV/Indels in Bile cfDNA

The gene mutations of bile cfDNA extracted by the 3D-BCF method were detected according to the next-generation sequencing technology, and the SNV/Indels of 10 genes were screened by bioinformatics analysis. These genes were verified by Sanger sequencing provided by Shanghai Personal Biotechnology Co., Ltd.

### Detection of Gene CNV in Bile cfDNA by FISH

According to the next-generation sequencing technology, the bile cfDNA extracted by the 3D-BCF method and the CNV mutations of three genes were determined by bioinformatics analysis. These genes were verified by FISH technology, and the testing service was provided by Shanghai Ackerman Medical Laboratory Co., Ltd.

### Statistics

Statistical analysis was performed using Prism 5 (GraphPad software). To detect the significant differences in the total amount of bile cfDNA extracted by each method, Two-way ANOVA (no matching) was used, and p < 0.05 indicated significant differences.

## Results

The full bile cfDNA extraction procedure is depicted in **Figure 1**. Bile samples were collected from patients using percutaneous transhepatic cholangial drainage (PTCD) before surgery (**Figure 1A**), and then pre-treated before cfDNA extraction. Bile was centrifuged at 1,600 g for 10 min followed by 16,000 g for 10 min at 4°C to remove all sediments, cellular components, and debris. The supernatants were stored in aliquots at -80°C before extraction. Silica-membrane-based spin-columns were used to enrich cfDNA in bile. After cfDNA was eluted from columns, Nanodrop, Qubit, and Agilent 2100 bioanalyzer were applied to perform cfDNA quality control, assess cfDNA purity, concentrations, and determine the size distributions of DNA fragments, respectively (**Figure 1B**).

### 3D-BCF Extraction Method Ability to Effectively Isolate DNA Fragments Spiked in Bile

**Figure 2:**
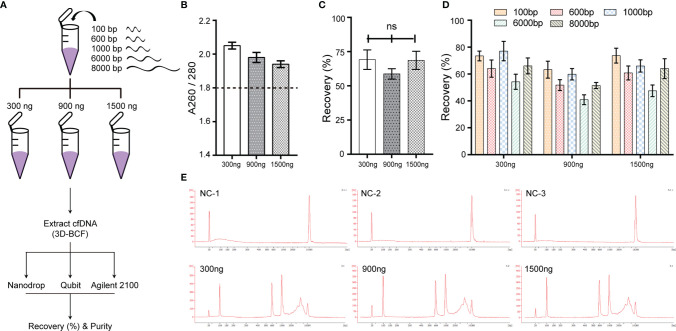
Extraction efficiency of the 3D-BCF method confirmed by spike-in assay. **(A)** Diagram of spike-in assay. Artificial DNA was spiked-in the solvent bile and then extracted by the 3D-BCF method. **(B)** Ratios of nucleic acids to proteins of DNA fragments extracted by 3D-BCF method. **(C)** Recovery rates of total spiked DNA fragments using 3D-BCF method. **(D)** Calculation of recovery rate of each extracted artificial DNA fragment using 3D-BCF method. **(E)** DNA fragment distribution of artificial DNA extracted by 3D-BCF method. ns, no significance.

To evaluate whether the proposed 3D-BCF extraction method could effectively isolate cfDNA from bile, fragmented DNA of lengths 100, 600, 1,000, 6,000, and 8,000 bps were equally mixed and spiked into 300-μL bile samples. Each bile sample was spiked with 300, 900, or 1,500 ng of total DNA amount (**Figure 2A**). Then, the 3D-BCF method was applied to extract the spiked DNA fragments from bile samples; Nanodrop, Qubit, and Agilent 2100 bioanalyzer were used to calculate the recovery rate of spiked DNA fragments. Little DNA could be extracted from the bile sample that served as solvent, but spiked DNA fragments could be successfully extracted, without bile protein pollution (A260/280 > 1.8, **Figure 2B**). Recovery rates in terms of total amount of DNA were calculated, showing 58.77% to 69.17% among all samples (**Figure 2C**). We calculated areas under the peaks of the DNA fragments and converted them to DNA amounts, and then compared those to the originally spiked-in amounts. Short DNA fragments (< 1,000 bps) exhibited very sharp peaks and better recovery rates at different DNA concentrations (**Figures 2D, E** **)**. The moderate recovery rates of long DNA fragments (6,000 and 8,000 bps) were probably due to the widening of the distribution peaks, and thus to the imprecision in calculating areas under the peaks of 6,000 and 8,000 bp DNA fragments (**Figure 2E**). These results indicate that the 3D-BCF method could effectively recover DNA fragments of different lengths from bile samples.

### Suitability of 3D-BCF Method for Bile cfDNA Extraction

**Figure 3:**
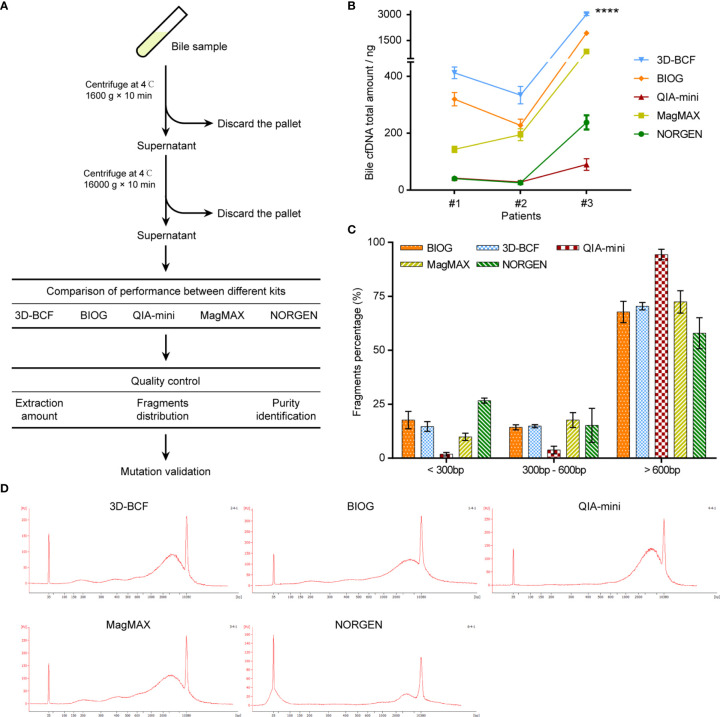
Comparison of five extraction methods in extracting cfDNA from patients-derived bile samples. **(A)** Flowchart of study design. Bile samples were derived from patients with malignant biliary tract tumors, and bile cfDNA was extracted using five different methods. **(B)** Extracted bile cfDNA amount obtained by different methods from three patient bile samples. **(C)** Percentages of different fragments of patient-derived bile cfDNA. **(D)** Representative images of fragment distributions of bile cfDNA derived from patients’ bile samples. ****, p < 0.0001.

We then applied the proposed method to bile samples from three patients, and compared the results with those from four other commercial cfDNA extraction kits (**Figure 3A**), namely the BIOG cfDNA Easy Kit (BIOG; Biogen), QIAamp DNA Mini Kit (QIA-mini; Qiagen), MagMAX^TM^ Cell-Free DNA Isolation Kit (MagMAX; Thermo Fisher), and Urine Cell-Free Circulating DNA Purification Mini Kit (NORGEN; Norgen Biotek). The proposed 3D-BCF method was able to extract the largest amount of cfDNA from all three patient bile samples, while BIOG extracted the second largest. The other three methods, i.e., QIA-mini, MagMAX, and NORGEN, were able to only extract much less bile cfDNA than 3D-BCF and BIOG (**Figure 3B**). Since plasma cfDNA and urine cfDNA were all highly fragmented, mostly under 200 bps in length, we also assessed bile cfDNA fragment distribution using the Agilent 2100 bioanalyzer (**Figures 3C, D** **)**. We determined the percentages of DNA fragment under 300 bps, 300–600 bps, and longer than 600 bps. Fragment distributions were similar in cfDNA extracted by the proposed 3D-BCF method and by BIOG, while QIA-mini lost most of the short fragments. NORGEN could extract more short fragments, but lost some long DNA fragments (**Figure 3C**). Representative images of bile cfDNA fragment distributions are shown in **Figure 3D**. All images of fragment distributions and the calculated percentages can be found in **Supplementary Figure 1**.

**Table 1** summarizes the characteristics of each method, including kit type and processing time for each sample, as well as the ability of extracting cfDNA and DNA quality. Although 3D-BCF and BIOG both have the ability to extract bile cfDNA of good quality, BIOG isolates less amount of cfDNA from bile samples than 3D-BCF. Taken together, 3D-BCF method is most suitable for bile cfDNA isolation based on the experimental results.

**Table 1:** Comprehensive comparison of different cfDNA extraction kits using bile samples of three patients with malignant tumors.

Bile cfDNA extraction method	Kit type	Time (min/single sample)	Patient	Avg. yield of total cfDNA ng/300μl bile	Avg. A260/A280 of bile cfDNA
**3D-BCF.**	Spin column	50	Patient #1	412.0 ± 20.4	1.77 ± 0.07
			Patient #2	334.0 ± 30.6	2.05 ± 0.04
			Patient #3	3018.0 ± 72.7	1.86 ± 0.01
**BIOG.**	Spin column	35	Patient #1	357.0 ± 67.0	1.86 ± 0.07
			Patient #2	254.6 ± 49.6	1.52 ± 0.27
			Patient #3	1924.0 ± 76.4	1.84 ± 0.01
**QIA-mini.**	Spin column	85	Patient #1	41.9 ± 1.5	1.87 ± 0.10
			Patient #2	27.9 ± 6.0	2.35 ± 0.58
			Patient #3	89.8 ± 20.5	2.11 ± 0.24
**MagMAX.**	Magnetic beads	70	Patient #1	143.0 ± 11.1	1.98 ± 0.09
			Patient #2	194.8 ± 20.9	1.99 ± 0.05
			Patient #3	862.0 ± 71.1	1.89 ± 0.03
**NORGEN.**	Spin column	40	Patient #1	40.4 ± 2.4	1.25 ± 0.02
			Patient #2	25.8 ± 4.8	1.00 ± 0.03
			Patient #3	237.8 ± 24.8	1.79 ± 0.01

Bile cfDNA extraction experiments of the five different methods were performed on bile samples derived from three different patients with malignant tumors. Each row represented the extraction results of each patient’s bile sample. The experiments were carried out in three replicates of each patient’s bile samples per method.

### 3D-BCF Extracted Bile cfDNA Carries Tumor-Related Genomic Information

**Figure 4:**
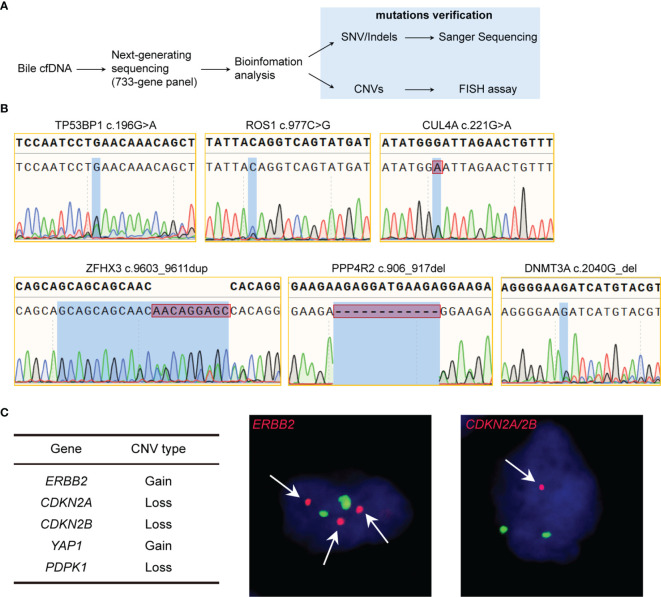
Validation of genomic alterations of extracted bile cfDNA. **(A)** Flowchart of validation of genomic alterations. **(B)** Sanger chromatograms of gene mutation sites. **(C)** Gene CNVs verified by FISH of tumor tissue. Red dots represents target gene probes, and green dots represent the centromeres of the chromosome where the target gene is located. Magnification 1000×.

Bile contacts tumors directly in biliary tract cancer patients, therefore, bile cfDNA is believed to contain tumor-related genomic alterations, serving as potential targets of liquid biopsy. To verify that the bile cfDNA extracted by the proposed 3D-BCF method contains BTCs genome information, we performed next-generation sequencing followed by site-specific Sanger sequencing and FISH assay for mutation verification (**Figure 4A**). A total of 47 nonsynonymous single nucleotide variations/insertions and deletions (SNV/Indels) in the coding sequence (CDS) regions of the 733-gene panel were found in the three bile cfDNA samples (coverage: 1252 ± 234 ×; **Supplementary Table 1**). Of these, ten SNV/Indels were selected and nine of them were successfully validated by Sanger sequencing following site-specific PCR (**Table 2**); designed primers and PCR conditions are listed in **Table 3**. Six representative mutations validated by PCR-Sanger sequencing are shown in **Figure 4B**, and others can be found in **Supplementary Figure 2**. *CUL4A* mutation (c.221G>A, MAF = 0.43) was clearly verified by PCR-Sanger sequencing, and heterozygous mutations in *TP53BP1* (c.196G>A, MAF = 0.21) and *ROS1* (c.977C>G, MAF = 0.44) also exhibited the obvious multiple peaks in Sanger chromatograms. *DNMT3A* c.2040G (MAF = 0.18) displayed heterozygous deletion, resulting in translational frame-shifting and mRNA nonsense-mediated decay. Short duplication in *ZFHX3* (c.9603_9611dup, MAF = 0.19) and short deletion in *PPP4R2* (c.906_917del, MAF = 0.27) were also apparent in Sanger chromatograms. *KRAS* c.38G>A (MAF = 0.02) failed to be verified, which may have been because of the low mutation frequency. In addition to SNV/Indels, copy number variations (CNVs) were found in five genes, consisting of copy number gains in *ERBB2* and *YAP1* and copy number losses in *CDKN2A*, *CDKN2B*, and *PDPK1*. Three variations were successfully verified by FISH experiment performed on tumor tissue specimens (*ERBB2*, *CDKN2A/2B*; **Figure 4C**). Taken together, these results show that the proposed method could efficiently extract cfDNA from bile, outperforming widely-used commercial kits. Moreover, the extracted bile cfDNA contains genomic information of biliary tract cancer patients, and could be applied to next generating sequencing, providing a potential means of using liquid biopsy to assist the early diagnosis of BTCs.

**Table 2:** SNVs of bile cfDNA verified by site-specific PCR and Sanger Sequencing.

Patient	Gene	Mutation Site	Type	MAF	RefSeq	Region	Consequence
**Patient #1**	*FAN1*	c.1137C>G	Nonsynonymous	0.20	NM_014967	CDS	p.F379L
	*TP53BP1*	c.196G>A	Nonsynonymous	0.21	NM_005657	CDS	p.E66K
	*ZFHX3*	c.9603_9611dup	Nonframeshift	0.19	NM_006885	CDS	p.Q3202_Q3204dup
	*NFE2L2*	c.1505G>A	Nonsynonymous	0.18	NM_006164	CDS	p.R502H
	*KRAS*	c.38G>A	Nonsynonymous	0.02	NM_004985	CDS	p.G13D
**Patient #2**	*CUL4A*	c.221G>A	Nonsynonymous	0.43	NM_003589	CDS	p.G74E
	*PPP4R2*	c.906_917del	Nonframeshift	0.27	NM_174907	CDS	p.D304_E307del
**Patient #3**	*DNMT3A*	c.2040del	Frameshift	0.18	NM_022552	CDS	p.I681Sfs*24
	*ROS1*	c.977C>G	Nonsynonymous	0.44	NM_002944	CDS	p.T326R
	*NOTCH4*	c.39_47dup	Nonframeshift	0.29	NM_004557	CDS	p.L14_L16dup

**Table 3:** Primers and PCR conditions for validation of mutation sites.

Patient	Target gene	Primer sequence	PCR condition	Amplicon size (bp)
**Patient #1**	*KRAS*	F: AAGCGTCGATGGAGGAGTTT	95°C 10 min, (95°C 20 s, 56°C 20 s, 72°C 40 s) × 35 cycles, 72°C 5 min	545
		R:TGGACCCTGACATACTCCCAA		
	*FAN1*	F: GAAAGCCAAAAGGCTACCCG	95°C 10 min, (95°C 20 s, 56°C 20 s, 72°C 40 s) × 35 cycles, 72°C 5 min	656
		R: CCCATGAACACTCCCACAGG		
	*TP53BP1*	F: CCATTCCAGGGGAGCAGATG	95°C 10 min, (95°C 20 s, 56°C 20 s, 72°C 40 s) × 35 cycles, 72°C 5 min	600
		R: ACGCAGATACCACAGTAGGC		
	*ZFHX3*	F: AGCCGAACTTGATGGGTCTG	95°C 10 min, (95°C 20 s, 56°C 20 s, 72°C 40 s)× 35 cycles, 72°C 5 min	415
		R: GCAGGGTCTACCGCATACTC		
	*NFE2L2*	F: CCTTGTCACCATCTCAGGGG	95°C 10 min, (95°C 20 s, 56°C 20 s, 72°C 40 s)× 35 cycles, 72°C 5 min	553
		R: TTGCCATCTCTTGTTTGCTGC		
**Patient #2**	*CUL4A*	F: ATACAAGGCGCGCTAGACTG	95°C 10 min, (95°C 20 s, 56°C 20 s, 72°C 40 s)× 35 cycles, 72°C 5 min	639
		R: AGCGTGTCATCTGGCTTTGT		
	*PPP4R2*	F: CGTATGGAAGACTTGCGGGT	95°C 10 min, (95°C 20 s, 56°C 20 s, 72°C 40 s)× 35 cycles, 72°C 5 min	1393
		R: ACCACTGATTTGCCCAGACC		
**Patient #3**	*DNMT3A*	F: AACGAGGCATGAGACAGAGC	95°C 10 min, (95°C 20 s, 56°C 20 s, 72°C 40 s)× 35 cycles, 72°C 5 min	693
		R: AACTGTCCAGAAACCAGCCC		
	*ROS1*	F: CATAACCCACTCTGGCCTGG	95°C 10 min, (95°C 20 s, 56°C 20 s, 72°C 40 s)× 35 cycles, 72°C 5 min	843
		R: CATGTTGGCAGCCTTCTTCG		
	*NOTCH4*	F:CCGAGGAGGAAGAAGAGGGG	95°C 10 min, (95°C 20 s, 56°C 20 s, 72°C 40 s)× 35 cycles, 72°C 5 min	300
		R: TCCATCCAGCATCCCTCACA		

## Discussion

BTCs, consisting of cholangiocarcinomas and gallbladder cancers, rank as the fifth most-frequent type of gastrointestinal malignancies in adults (18). BTCs are usually asymptomatic in early stages, and are often diagnosed at advanced stages when tumors have already metastasized and patients have missed the optimal opportunity for surgery (19). The median overall survival of BTC patients is less than 1 year, and the 5-year survival rate of patients at late stages is less than 10% (20, 21). The diagnosis of BTCs mainly relies on serum biomarkers, radiographic imagings, and endoscopic examinations. However, the complex anatomic structure of the hepatobiliary system compromises the accuracy of radiography diagnosis, and remarkably increases the difficulty of tissue biopsies. Therefore, devising a convenient approach with high accuracy in diagnosing BTCs is quite urgent.

Our previous study revealed that bile contains an abundance of cfDNA, which carries tumor genomic information that could help identify targets for liquid biopsies (12). However, there is no established method or commercial kit for bile cfDNA extraction. Our goal was to establish an extraction method and a quality control system suitable for bile cfDNA. The kit most commonly reported as being used for bile cfDNA extraction at the time we began this study was the QIAamp Circulating Nucleic Acid Kit, which is designed for plasma cfDNA isolation and delivers excellent recovery of fragmented nucleic acids as short as 75 bps (15). Nevertheless, in our previous study, we found that unlike plasma or urine cfDNA which is highly fragmented, large fragments were prevalent in bile cfDNA (12). In this study, we selected a silica membrane that was able to recover DNA as short as 40 bp and as long as 10 kb for bile cfDNA extraction. To verify its ability to extract DNA from bile, DNA fragments of different lengths were spiked into the bile sample. 100-, 600-, and 1 000-bp DNA fragments did not reveal any significant difference in recovery rate, while 6,000- and 8,000-bp DNA fragments showed a relatively lower recovery rate (**Figure 2D**). DNA fragments of lengths less than 1,000 bp exhibited specific sharp peaks in the Agilent 2100 bioanalyzer, but 6,000- and 8,000-bp DNA fragments exhibited obviously widened peaks (**Figure 2E**), which may be caused by the harsh physiochemical conditions of bile for long DNA fragments to maintain their double-helix structure. Given the complexity of bile components, the proposed method could reach a recovery rate of approximately 70%. This rate is quite satisfying since the best performance reported for commercial kits with respect to isolating cfDNA from plasma is over 80% recovery (15), and that for isolating cfDNA from urine is 73%–84% recovery (22).

Of the five methods used in this study, the BIOG kit showed closest similarity to the proposed 3D-BCF method in bile cfDNA extraction (**Figure 3**). This kit is also based on the use of a silica membrane to capture DNA. cfDNA yields using the BIOG method are slightly lower than those using the 3D-BCF method, while both small and large DNA fragments in bile are efficiently collected. The NORGEN kit is designed for isolating urine cfDNA, the fragments of which are even shorter than those of plasma cfDNA, and is supplied with hybrid silica/silicon carbide spin columns specially designed for short fragments in urine. The NORGEN kit reveals the highest recovery rate of short DNA fragments, in accordance with superior capture of ultrashort fragments claimed by the manufacturer. However, its ability to capture DNA fragments longer than 600 bps is limited. Unfortunately, Oreskovic et al. reported that NORGEN could led to consistent PCR inhibition (22), which may cause dismal output of library construction for sequencing. The QIA-mini kit is another kit that uses silica membrane based spin columns, and it produces DNA of up to 50 kb in size. The QIA-mini kit is designed for genomic DNA purification from various tissues since genomic DNA fragments are extremely long, and we wonder whether long-fragment bile cfDNA could be efficiently isolated. To our surprise, the QIA-mini kit extracted the least bile cfDNA among all of the methods, with only a small amount of short (< 300 bps) or medium (300–600 bps) DNA able that could be purified (**Figure 3D**). Although QIAamp Circulating Nucleic Acid Kit is also based on silica membranes, and is identified as one of the most suitable options for plasma cfDNA (23, 24), pronounced differences between these two commercial kits are clearly visible. The MagMAX kit depends on pre-concentration of cfDNA onto magnetic beads. It has been reported to have high recovery of longer fragments but very low detectable recovery for short fragments in urine (22). We found that this kit was able to purify both long and short DNA fragments from bile, but the recovery rate was moderate. In addition, the time consumed by this kit was the second longest (**Table 1**). Our results reveal that the proposed 3D-BCF method is most suitable for bile cfDNA extraction, exhibiting the best performance in isolating cfDNA from patient bile samples with moderate experimental time consumption per sample.

In addition to cfDNA, bile has also been a source of RNAs or extracellular vesicles (EVs) for detecting BTCs. The miRNA profiles of patients with cholangiocarcinoma are distinct from those of patients with primary sclerosing cholangitis (25). miR-30d-5p in bile showed a diagnostic performance with a sensitivity of 81.1% and a specificity of 60.5% when discriminating cholangiocarcinoma patients from patients with benign biliary diseases (26). EVs are also present exists in bile, and human bile EVs contain abundant miRNAs and proteins that can be used to develop disease marker panels for biliary tract cancers (27, 28). The expression of miR-483-5p and miR-126-3p was found to be significantly higher in bile EVs derived from patients with malignant biliary obstructions, and could distinguish benign and malignant biliary obstructions with high accuracy and specificity (29). Claudin-3, a protein derived from human bile EVs sample, was identified as cholangiocarcinoma-specific, and the diagnostic accuracy was 0.945 AUC (30). Besides, long non-coding RNAs from bile EVs, ENST00000588480.1 and ENST00000517758.1 also showed significantly up-regulated expression in patients with cholangiocarcinoma. The higher expression levels of the two lncRNAs were significantly associated with poor survival, indicating the bile EVs lncRNAs as potential diagnostic and therapeutic targets (31). In summary, bile serves as a good source of tumor biomarkers including cfDNAs, miRNAs, and EVs, for the detection of biliary tract cancers, and the 3D-BCF method we established may assist in identifying bile cfDNA as a promising liquid biopsy for BTCs patients.

There are several limitations worth mentioning in this study. First, bile samples were collected from three biliary tract cancer patients, composed of one gallbladder cancer and two perihilar cholangiocarcinomas. For comparison of extraction efficiency, increasing the number of patients would provide more statistical power. A future study with a greater number of patients may provide confirmation of this study. Second, the selected commercial kits includes different types, such as blood/tissue DNA isolation and urine cfDNA isolation, and can also be categorized into spin-column based or magnetic-beads based methods. There are many other kinds of DNA isolation kits, which may be used for further comparison to identify the most suitable method for bile cfDNA isolation. Finally, PCR-Sanger chromatograms, which rely heavily on MAF, were used for validation of mutations detected by NGS. Mutations with a MAF of less than 10% were almost undetectable and validated by PCR-Sanger chromatograms. Techniques with greater sensitivity and accuracy in detecting gene mutations, such as ddPCR, could be used for SNV/Indels verification in the next phase of our research.

## Conclusion

Bile cfDNA could provide a “power tool” for BTC patients to benefit from liquid biopsy, making early diagnosis and better prognosis possible. Nevertheless, how to effectively isolate cfDNA of good quality from bile remains to be explored. In the present work, we established an extraction method for bile cfDNA, and confirmed its high recovery efficiency by a spike-in assay and its advantages in bile cfDNA isolation through comparisons with other commercial nucleic acid isolation kits. We also performed next-generation sequencing on the extracted bile cfDNA, and verified the detected mutation sites and CNVs by Sanger chromatograms and FISH assay. In summary, the proposed 3D-BCF method could efficiently extract bile cfDNA with good quality, providing technical support for bile-based liquid biopsy in managing BTC patients.

## Data Availability Statement

The original contributions presented in the study are included in the article/**Supplementary Material**. Further inquiries can be directed to the corresponding authors.

## Ethics Statement

The studies involving human participants were reviewed and approved by Ethics Committee of the Shanghai Eastern Hepatobiliary Surgery Hospital, Second Military Medical University/Navy Medical University (EHCHKY2018-K-003). The patients/participants provided their written informed consent to participate in this study.

## Author Contributions

YZ, XF, and DZ performed study concept and design; NS, BZ, BN, XX, SC, and ZL performed the experiments; WZ, BN, XC, and XW analysed the data; NS, BZ, LY, XR, YL, XS, XH, CC, and LX provided technical and material support; WZ, BN, and DZ performed development of methodology and writing, review and revision of the paper. All authors read and approved the final manuscript.

## Funding

This work was supported by Shanghai Sailing Program (No. 21YF1458500).

## Conflict of Interest

Authors WZ, BN, XX, SC, XC, ZL, CC, LX, and DZ are/were employed by 3D Medicines Inc.

The remaining authors declare that the research was conducted in the absence of any commercial or financial relationships that could be construed as a potential conflict of interest.

## Publisher’s Note

All claims expressed in this article are solely those of the authors and do not necessarily represent those of their affiliated organizations, or those of the publisher, the editors and the reviewers. Any product that may be evaluated in this article, or claim that may be made by its manufacturer, is not guaranteed or endorsed by the publisher.

## Supplementary Material

Supplementary Figure 1 Fragment distributions of every bile cfDNA sample. **(A)** Bile cfDNA fragment distributions of every sample. **(B)** Calculation of bile cfDNA percentages of each patient.

Supplementary Figure 2 Sanger chromatograms of selected gene SNV/Indels.

Supplementary Table 1 SNV/Indels of bile cfDNA.
