# Supplementary figures and images for: Comprehensive Evaluation and Application of a Novel Method to Isolate Cell-Free DNA Derived From Bile of Biliary Tract Cancer Patients
Source: Front Oncol. 2022 May 4;12:891917. doi: 10.3389/fonc.2022.891917 (PMC9116272; doi:10.3389/fonc.2022.891917)

FIGURE S1

A

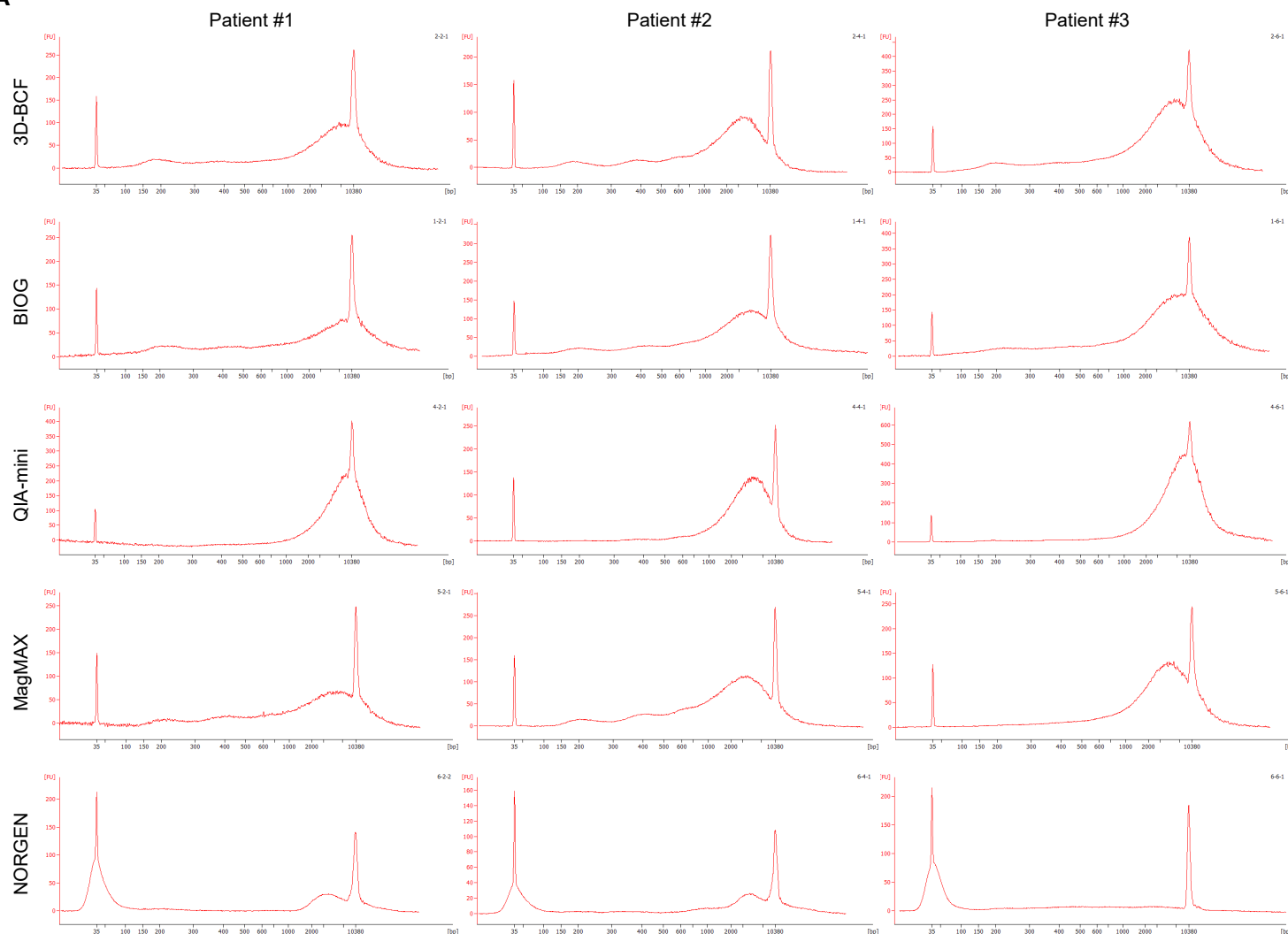

B

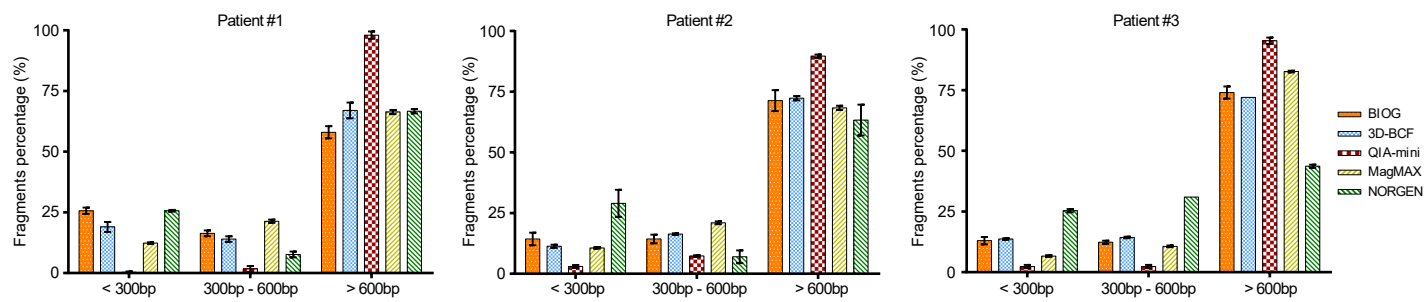

Supplement: Supplementary Figure 1 — Fragment distributions of every bile cfDNA sample. (A) Bile cfDNA fragment distributions of every sample. (B) Calculation of bile cfDNA percentages of each patient. [file DataSheet_1.pdf]

FIGURE S2

FAN1 c.1137C>G

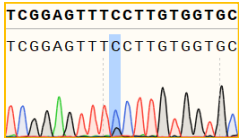

NFE2L2 c.1505G>A

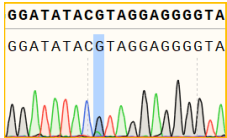

NOTCH4 c.39\_47dup

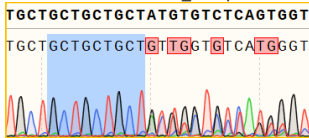

Supplement: Supplementary Figure 2 — Sanger chromatograms of selected gene SNV/Indels. [file DataSheet_2.pdf]
