# Supplementary material for: Comprehensive Evaluation and Application of a Novel Method to Isolate Cell-Free DNA Derived From Bile of Biliary Tract Cancer Patients
Source: Front Oncol. 2022 May 4;12:891917. doi: 10.3389/fonc.2022.891917 (PMC9116272; doi:10.3389/fonc.2022.891917)
Supplement: Supplementary Table 1 — SNV/Indels of bile cfDNA. [file DataSheet_3.pdf]

Table S1. SNV/Indels of bile cfDNA.

SampleID	chr	chr_start	chr_end	reference_allele	variant_allele	MAF	coverage	vsr	cdot	gene	type	refseq	region
Patient #1	chr12	25398281	25398281	C	T	0.02	836	17	c.38G>A	KRAS	Nonsynonymous	NM_004985	CDS
Patient #1	chrX	152825336	152825336	G	C	0.06	941	52	c.2775G>C	ATP2B3	Nonsynonymous	NM_021949	CDS
Patient #1	chr8	68864693	68864693	C	T	0.13	949	120	c.64C>T	PREX2	Nonsynonymous	NM_024870	CDS
Patient #1	chr2	178095826	178095826	C	T	0.18	709	125	c.1505G>A	NFE2L2	Nonsynonymous	NM_006164	CDS
Patient #1	chr16	72822563	72822563	-	TGCTGCTGC	0.19	1452	275	c.9603_9611dup	ZFHX3	Nonframeshift	NM_006885	CDS
Patient #1	chr15	31198003	31198003	C	G	0.20	1472	289	c.1137C>G	FAN1	Nonsynonymous	NM_014967	CDS
Patient #1	chr15	43784275	43784275	C	T	0.21	925	192	c.196G>A	TP53BP1	Nonsynonymous	NM_005657	CDS
Patient #1	chr12	124810908	124810908	C	T	0.43	956	412	c.7192G>A	NCOR2	Nonsynonymous	NM_006312	CDS
Patient #1	chr17	59793411	59793411	C	T	0.45	573	257	c.2393G>A	BRIP1	Nonsynonymous	NM_032043	CDS
Patient #1	chr15	89868853	89868853	T	C	0.46	1075	490	c.1777A>G	POLG	Nonsynonymous	NM_002693	CDS
Patient #1	chr6	18130918	18130918	T	C	0.47	562	262	c.719A>G	TPMT	Nonsynonymous	NM_000367	CDS
Patient #1	chr2	234669144	234669144	G	A	0.48	1473	710	c.211G>A	UGT1A1	Nonsynonymous	NM_000463	CDS
Patient #1	chr6	31725978	31725978	C	G	0.50	988	491	c.1051C>G	MSH5	Nonsynonymous	NM_172165	CDS
Patient #2	chr20	31023462	31023480	CTGAAAATCAACGGAGACT	-	0.03	1488	50	c.2952_2970del	ASXL1	Frameshift	NM_015338	CDS
Patient #2	chr2	29462550	29462550	G	C	0.04	1338	54	c.2351C>G	ALK	Nonsynonymous	NM_004304	CDS
Patient #2	chr20	31022762	31022762	C	-	0.05	1599	72	c.2250del	ASXL1	Frameshift	NM_015338	CDS
Patient #2	chr5	131944976	131944976	A	T	0.05	502	26	c.2924A>T	RAD50	Nonsynonymous	NM_005732	CDS
Patient #2	chr3	73114255	73114266	AGAAGAGGATGA	-	0.27	583	156	c.906_917del	PPP4R2	Nonframeshift	NM_174907	CDS
Patient #2	chr13	113887499	113887499	G	A	0.43	577	246	c.221G>A	CUL4A	Nonsynonymous	NM_003589	CDS
Patient #2	chr6	111628734	111628734	C	T	0.45	522	233	c.9082G>A	REV3L	Nonsynonymous	NM_002912	CDS
Patient #2	chr10	115612610	115612610	G	T	0.45	1233	556	c.332C>A	DCLRE1A	Nonsynonymous	NM_014881	CDS
Patient #2	chr15	74336952	74336952	G	C	0.46	1657	770	c.2252G>C	PML	Nonsynonymous	NM_033238	CDS
Patient #2	chr2	128050268	128050268	C	T	0.47	1126	526	c.389G>A	ERCC3	Nonsynonymous	NM_000122	CDS
Patient #2	chr12	56818580	56818580	C	T	0.47	998	472	c.1834G>A	TIMELESS	Nonsynonymous	NM_003920	CDS
Patient #2	chr6	94066470	94066470	A	G	0.48	728	351	c.1289T>C	EPHA7	Nonsynonymous	NM_004440	CDS
Patient #2	chr16	56871605	56871605	G	A	0.49	982	478	c.1985G>A	NUP93	Nonsynonymous	NM_014669	CDS
Patient #2	chr2	100623366	100623366	C	G	0.49	1132	558	c.676G>C	AFF3	Nonsynonymous	NM_001025108	CDS
Patient #2	chr9	139391403	139391403	C	T	0.49	1275	629	c.6788G>A	NOTCH1	Nonsynonymous	NM_017617	CDS
Patient #2	chr9	98003002	98003002	A	G	0.50	522	259	c.274T>C	FANCC	Nonsynonymous	NM_000136	CDS
Patient #2	chr19	17953321	17953321	C	T	0.50	825	410	c.665G>A	JAK3	Nonsynonymous	NM_000215	CDS
Patient #2	chr16	3640653	3640653	C	G	0.50	1661	832	c.2986G>C	SLX4	Nonsynonymous	NM_032444	CDS
Patient #2	chr16	1825869	1825869	G	A	0.51	1318	667	c.851G>A	EME2	Nonsynonymous	NM_001257370	CDS
Patient #2	chr6	106554373	106554373	A	G	0.53	943	498	c.1901A>G	PRDM1	Nonsynonymous	NM_001198	CDS
Patient #2	chr19	10476226	10476226	C	T	0.53	1414	749	c.978G>A	TYK2	Stopgain	NM_003331	CDS
Patient #2	chr17	41246083	41246083	C	A	0.54	1215	658	c.1465G>T	BRCA1	Stopgain	NM_007294	CDS
Patient #3	chr16	72821593	72821593	-	GCCGCC	0.17	1753	290	c.10576_10581dup	ZFHX3	Nonframeshift	NM_006885	CDS
Patient #3	chr2	25464473	25464473	C	-	0.18	1972	347	c.2040del	DNMT3A	Frameshift	NM_022552	CDS
Patient #3	chr6	32191658	32191658	-	AGCAGCAGC	0.29	1914	555	c.39_47dup	NOTCH4	Nonframeshift	NM_004557	CDS
Patient #3	chr6	117715781	117715781	G	C	0.44	864	381	c.977C>G	ROS1	Nonsynonymous	NM_002944	CDS
Patient #3	chr11	94194169	94194169	T	G	0.45	859	383	c.1259A>C	MRE11	Nonsynonymous	NM_005591	CDS
Patient #3	chr6	31138310	31138310	C	T	0.45	2178	975	c.88G>A	POU5F1	Nonsynonymous	NM_002701	CDS
Patient #3	chr13	32930651	32930651	G	A	0.47	1570	737	c.7522G>A	BRCA2	Nonsynonymous	NM_000059	CDS
Patient #3	chr19	54652382	54652382	C	T	0.48	1946	929	c.1310C>T	CNOT3	Nonsynonymous	NM_014516	CDS
Patient #3	chr15	89864042	89864042	G	A	0.48	1495	716	c.2936C>T	POLG	Nonsynonymous	NM_002693	CDS
Patient #3	chr4	178283479	178283479	A	C	0.48	801	386	c.1672A>C	NEIL3	Nonsynonymous	NM_018248	CDS
Patient #3	chr12	124907096	124907096	A	G	0.49	1629	801	c.1334T>C	NCOR2	Nonsynonymous	NM_006312	CDS
Patient #3	chr11	128786525	128786525	G	C	0.50	1725	860	c.1159G>C	KCNJ5	Nonsynonymous	NM_000890	CDS
